# *Indica* and *Japonica* Crosses Resulting in Linkage Block and Recombination Suppression on Rice Chromosome 12

**DOI:** 10.1371/journal.pone.0043066

**Published:** 2012-08-17

**Authors:** Yulin Jia, Melissa H. Jia, Xueyan Wang, Guangjie Liu

**Affiliations:** 1 Dale Bumpers National Rice Research Center, Agricultural Research Service, United States Department of Agriculture, Stuttgart, Arkansas, United States of America; 2 Department of Biology, College of Life Sciences, China Jiliang University, Hangzhou, Zhejiang, China; 3 Rice Research and Extension Center, University of Arkansas, Stuttgart, Arkansas, United States of America; New Mexico State University, United States of America

## Abstract

Understanding linkage block size and molecular mechanisms of recombination suppression is important for plant breeding. Previously large linkage blocks ranging from 14 megabases to 27 megabases were observed around the rice blast resistance gene *Pi-ta* in rice cultivars and backcross progeny involving an *indica* and *japonica* cross. In the present study, the same linkage block was further examined in 456 random recombinant individuals of rice involving 5 crosses ranging from F_2_ to F_10_ generation, with and without *Pi-ta* containing genomic *indica* regions with both *indica* and *japonica* germplasm. Simple sequence repeat markers spanning the entire chromosome 12 were used to detect recombination break points and to delimit physical size of linkage blocks. Large linkage blocks ranging from 4.1 megabases to 10 megabases were predicted from recombinant individuals involving genomic regions of *indica* and *japonica*. However, a significantly reduced block from less than 800 kb to 2.1megabases was identified from crosses of *indica* with *indica* rice regardless of the existence of *Pi-ta*. These findings suggest that crosses of *indica* and *japonica* rice have significant recombination suppression near the centromere on chromosome 12.

## Introduction

Recombination suppression is a complex biological phenomenon often resulting in large linkage blocks (LBs) where a set of genes is inherited together. Recombination suppression is known to be determined by the location of a genomic region, the method of selection, and genetic factors that determine recombination [1], [2]. Although it is known that recombination suppression can easily occur in regions with more repetitive DNA sequences and/or near the centromere regions [3], [4], [5], [6], the molecular and genetic bases of recombination suppression have not been clearly defined.

Rice (*Oryza sativa* L.) is one of the most important food crops in the world. Blast disease of rice caused by the filamentous fungal pathogen *Magnaporthe oryzae* has been a serious threat to rice production for years in most regions where rice is grown. Breeding for resistance to blast has been one of the most important objectives for rice breeders worldwide. Over 80 major and 35 quantitative *R* genes have been incorporated, some individually and/or in clusters, into diverse elite cultivars by classical plant breeding [7]. In practice, resistant resources for *japonica* rice growing areas have been commonly identified from subspecies *indica* whereas *japonica* resistant resources are useful for *indica* rice growing areas [8]. Crosses between *indica* and/or *japonica* rice have been routinely made for incorporating these blast *R* genes. The major *R* gene *Pi-ta* in rice was introduced from landrace *indica* varieties, Tetep and Tadukan, into a series of *japonica* cultivars in the Southern US and other regions of the world. A total of 11 newly developed rice cultivars, Katy, Drew, Madison, Kaybonnet, Banks, Ahrent, Spring, Cybonnet, Catahoula, CL111, and Templeton, were confirmed to contain *Pi-ta* [9]. *Pi-ta* encodes a cytoplasmic receptor with nucleotide binding domain and imperfect leucine rich repeats preventing infections by races of *M. oryzae* that contain *AVR-Pita* [10]. *AVR-Pita,* known to encode a metalloprotease, was predicted to be involved in pathogenicity [11]. Distribution, evolution and interaction of *Pi-ta* and *AVR-Pita1* have been investigated [12], [13], [14], [15], [16], [17], [18], [19], [20], [21]. As a result, DNA markers have been identified from portions of the cloned gene for marker assisted selection [22], [23]. In a previous study, large LBs around the *Pi-ta* locus were identified from backcrossed progeny and several rice cultivars [24]. It was unclear if the observed large LBs were due to multiple genes required for *Pi-ta* mediated resistance that is clustered within the same genomic region [25] and/or were due to recombination suppression because of incompatibility between *indica* and *japonica*.

The objective of the present study was to examine LBs at the *Pi-ta* locus using five random mapping populations involved in recombination events of *indica* with *japonica* and *indica* with *indica*.

## Results

### LB between a Cross of a *japonica* Line (*pi-ta/pi-ta*) with a *japonica* Cultivar Carrying a Portion of *indica* Genome (*Pi-ta/Pi-ta*)

**Figure 1:**
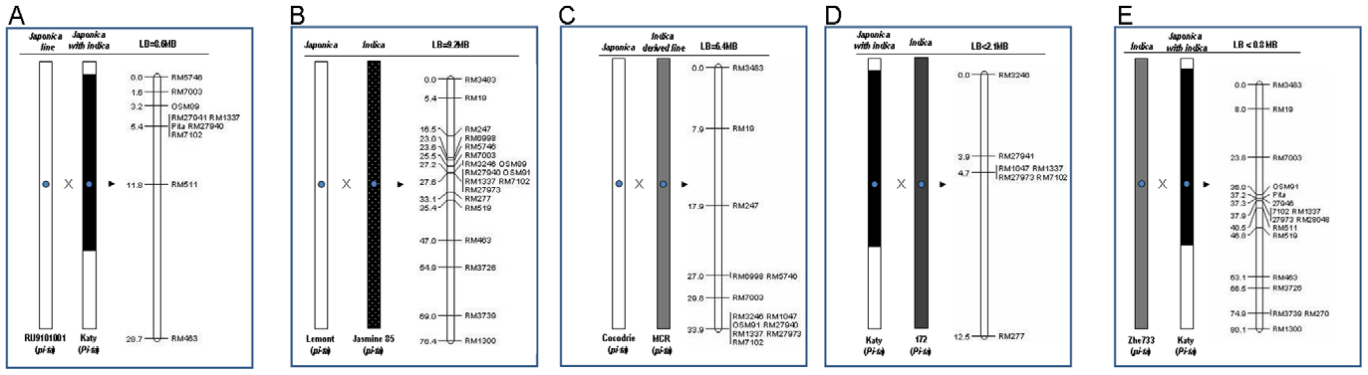
Genetic maps showing the size of linkage block among different crosses. A: Lemont and Jasmine 85; B: Early and Katy; C. Cocodrie and MCR 01-0277; D. Katy and Amane (172); E: Zhe733 and Katy. Graphic presentation of chromosome 12 with centromere, and difference of genotypes were indicated by different color shading (Left). Genetic distance in CentiMorgan defined by SSR marker and physical size of the linkage block defined by the two closest SSR markers was shown as LB = (Right) were shown.

To further investigate the recombination suppression observed at the *Pi-ta* locus in germplasm selected for blast resistance [24], we investigated a random RIL population consisting of 87 lines or 174 recombination events of a *japonica* breeding line RU9101001 (Early) with a *japonica* cultivar Katy with a large fragment of *Pi-ta* containing *indica* genome on chromosome 12. Markers RM27941, RM1337, RM27940 and RM7102 co-segregated with *Pi-ta*. SSR marker OSM89 physically located at 8.8 megabases (MB) was found at the left border at 2.2 cM from the *Pi-ta* gene; whereas, SSR marker RM511 physically located at 17.4 MB was found at the right border 6.4 cM from the *Pi-ta* gene. LB in this population was reduced to 8.6 MB (Figure 1A).

### LBs between Crosses of Two *japonica* Cultivars (*pi-ta/pi-ta*) with Two *indica* Cultivars (*pi-ta/pi-ta*)

Analysis of 186 recombinant events from Lemont (*pi-ta/pi-ta*) and Jasmine 85 (*pi-ta/pi-ta*) RIL population identified SSR markers OSM91 and RM7102 that were found to co-segregate perfectly with *Pi-ta*. The physical distance between these two markers is 3.1 MB (Table 1). However, SSR marker RM3246, located at 9.1 MB, was identified at 0.4 cM from the *Pi-ta* gene that defines the left border; whereas SSR marker RM277 located at 18.3 MB is 5.5 cM from *Pi-ta* that defines the right border (Figure 1B). These two border markers indicated an LB of 9.2 MB.

**Table 1:** Physical distances of SSR markers co-segregated with *Pi-ta* and with both borders of the recombination break points in mapping populations.

	Left border		Co-segregated		Right border	
	Marker	Physicallocation (MB)^1^	Marker	Physical distance between two markers (MB)^2^	Marker	Physical location
Lemont X Jasmine 85	RM3246	9.1	OSM91-RM7102	3.1	RM277	18.3
RU9101001 X Katy	OSM89	8.8	*Pi-ta*-RM7102	2.6	RM511	17.4
Zhe733 X Katy	OSM91	10.1	*Pi-ta*	0	RM 27946	10.9
Cocodrie X MCR	RM7003	6.8	RM3246-RM7102	4.1	None	
Katy X 172	RM3246	9.1	RM27941	0	RM1047	11.2

1 Physical location of each marker was obtained from www.gramene.org.

2 MB denotes megabases.

A population derived from a cross of a different *pi-ta/pi-ta indica* cultivar with a different *pi-ta/pi-ta japonica* cultivar was studied to determine if LB can be reduced if a different *indica* genome on chromosome 12 is used. A total of 93 random doubled haploid lines or 186 recombination events of a tropical *japonica* cultivar Cocodrie (*pi-ta/pi-ta*) and an *indica* derived germplasm MCR 01-0277 (*pi-ta/pi-ta*) were analyzed. SSR marker RM7003 physically located at 6.8 MB was found at the left border of the *pi-ta* gene. RM3246, RM1047, OSM91, RM27940, RM1337, RM27973 and RM7102, the furthest marker whose physical location is 13.2 MB, co-segregated with *pi-ta*. This analysis identified a LB is greater than 6.4 MB (Figure 1C). Unfortunately, the exact LB block was undetermined because we failed to identify the right border in this population. This finding suggests that observed linkage block is largely due to genomic contents on chromosome 12.

### LB between a Cross of a *japonica* Cultivar Carrying *Pi-ta indica* Region with an *indica* Cultivar with *Pi-ta* and a Defective Component

To investigate if LB can be reduced in germplasm containing *Pi-ta* from two different origins, a total of 94 individuals (188 recombination events) of an F_2_ population derived from a cross of Katy (*Pi-ta/Pi-ta*) with an *indica* cultivar 172 from Sri Lanka (*Pi-ta/Pi-ta*) but missing additional critical components for blast resistance where both contain different *indica* origins at chromosome 12 were analyzed. SSR marker RM3246 whose physical location is 9.1 MB was identified at 1.2 cM from the *Pi-ta* gene. SSR marker RM27941 co-segregated with *Pi-ta*. The SSR marker RM1047 whose physical location is 11.2 MB was identified at 0.8 cM from the *Pi-ta* gene defining the right border. LB was further delimited within a 2.1 MB fragment (Figure 1D).

### LB between a Cross of *japonica* Carrying an *indica* Genome at the *Pi-ta* Locus with an *indica* Cultivar (*pi-ta/pi-ta*)

We investigated if LB can be reduced when a *japonica* cultivar carrying *indica* genome on chromosome 12 containing *Pi-ta* was crossed with an *indica* cultivar from a different rice growing area lacking *Pi-ta.* A total of 89 individuals of an F_10_ RIL population of a cross of an *indica* Zhe733 (*Pi-ta/Pi-ta*) and tropical genetic stock Kaybonnet*-lpa* (*Pi-ta/Pi-ta*) with chromosome 12 identical to that in Katy was analyzed (Table 1, Figure 1). SSR marker OSM91 (10.1 MB) was identified at 1.2 cM from the *Pi-ta* gene and RM27946 (10.9 MB) was identified 0.1 cM at the right border of the *Pi-ta* gene. This analysis further determined that LB was independent to *Pi-ta*, and was within 0.8 MB (Figure 1E).

## Discussion

**Figure 2:**
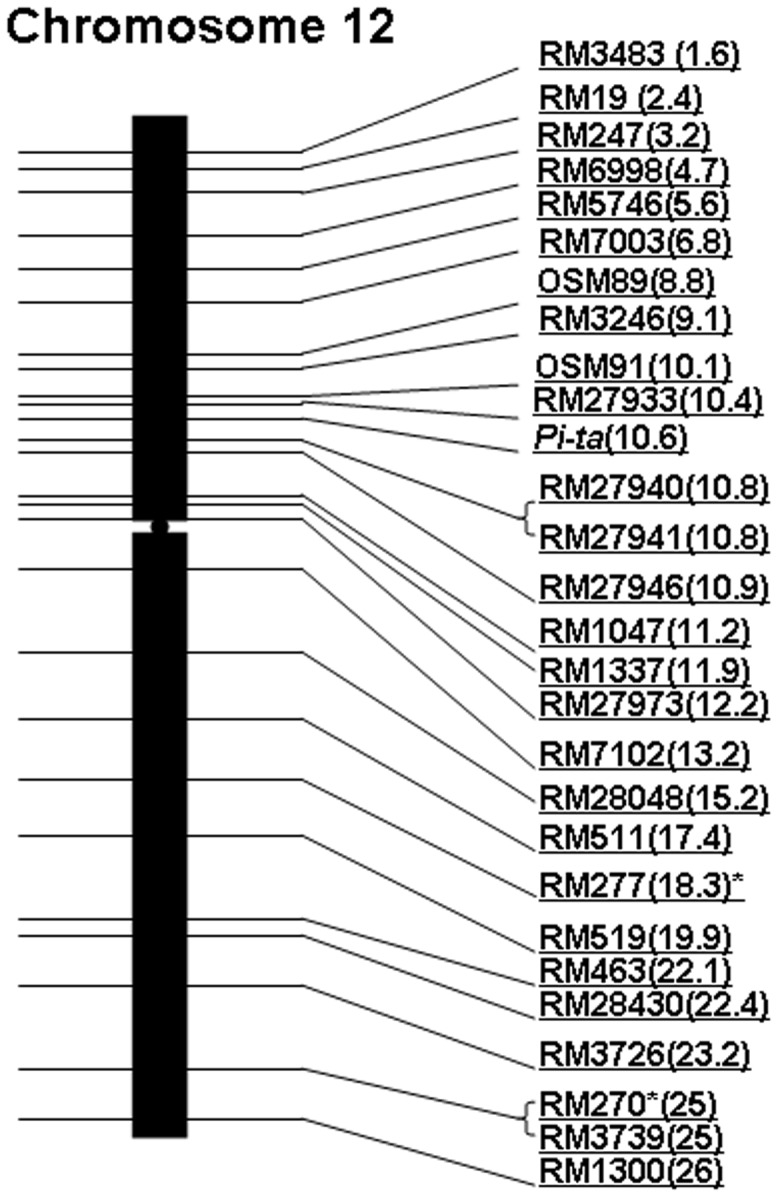
Schematic presentation of chromosome 12 with physical location (MB) of each SSR marker used for this study. Locations of SSR markers were obtained from www.gramene.org. *Physical location unavailable in gramene thus was determined by blasting the primers to the Nipponbare genome.

Investigation of the molecular and genetic basis of LB and recombination suppression can benefit plant breeding and genetic studies. In a previous study, we demonstrated a large linkage block from 43 BC_5_F_2_ progeny and several elite rice cultivars around the rice blast resistance gene *Pi-ta* [24]. It was proposed that selection for genes required for blast resistance could be one of the major reasons for the observed linkage blocks. In the present study, we examined if the observed linkage block could also be due to the incompatibility between *indica* and *japonica* in crosses without selection pressure for blast resistance. A total of 5 random recombinant populations consisting of 456 individuals with different resistant and susceptible *Pi-ta* haplotypes were examined using 40 SSR markers spanning the centromeric region of chromosome 12 (Figure 2). Remarkably, differential recombinant events between individuals derived from japonica and indica, indica and indica crosses were detected, LB was delimited within 800 KB between the *indica* cultivar Zhe733 without the resistant *Pi-ta* allele (Figure 1E), and a tropical *japonica* cultivar Katy carrying resistant *indica Pi-ta* allele from an *indica* landrace variety Tetep; whereas a large 9.2 MB linkage block was observed between a *japonica* lacking *Pi-ta* and *indica* containing *Pi-ta* and further reduced to be 6.4 MB with *japonica* and *indica* derived lines lacking the resistant *Pi-ta* allele. Taken together, this study suggests that the existence of a large linkage block at the centromeric region at the *Pi-ta* locus is mostly due to incompatibility between *indica* and *japonica*. To our knowledge this is the first demonstration of this magnitude of lack of recombination between *indica* and *japonica* rice crosses involving chromosome 12.

We knew that an individual of an F2 or DH population has less recombination than an individual of a RIL population. In the present study, three RIL populations were used to investigate the magnitude of LBs. Of the three RIL populations studied two were indica by japonica, and one was indica by indica to ensure similar recombination rates. It was found that indica by indica RIL population had significantly higher recombination rates than indica by japonica crosses. We might have observed an even smaller LB in the Katy, a japonica cultivar with large indica genomic region by an indica cultivar “172” mapping population if it had been advanced to RIL generation. With less recombination events we still detected 2.1 MB LB that is smaller than other indica and japonica crosses and larger than other indica and indica cross. In the present study, the numbers of individuals ranged from 87–93 in 5 mapping populations were comparable. While these populations were slightly small, it’s highly improbable that we would observe large LBs in three different indica by japonica crosses while not observing it in two indica by indica crosses. Significant reduction of LB from indica and japonica cross in comparison with that of indica and indica cross suggests that large LB could be due to indica and japonica cross. Further examination of LBs from japonica and japonica cross will help to understand the genetic and molecular bases of LB.

The existence of sexual incompatibility between *indica* and *japonica* rice on chromosome 12 suggest that both *japonica* and *indica* are not as closely related subspecies of cultivated rice *Oryza sativa* as previously thought. It is well known that *japonica* and *indica rice* possess distinct morphological and molecular differences [26] suggesting unique origins and domestications. One theory is that *O. sativa* was originated from the wild relative *Oryza rufipogon.* However, historical records of the time and geographic separation of *indica* from *japonica* subspecies remains unclear. Furthermore, Xu et al [27] reported sequence information of cultivars and wild relatives of rice suggesting that *indica* is more closely related with wild relative *O. nivara* and *japonica* is closely related with *O. rufipogon* [27]. Contemporary surveys have indicated that *indica* rice is commonly grown in lowland throughout tropical Asia while *japonica* is often cultivated in dry fields in temperate East Asia, upland areas of Southeast Asia and high elevations in South Asia, and the United States of America (US). There is also another theory that *Oryza sativa* rice was domesticated at least twice where *indica* was from eastern India, Myanmar and Thailand; and *japonica* from southern China and Vietnam even though there is archaeological and genetic evidence for a single domestication of rice from the lowlands of China [28], [29]. How environmental conditions and domestication contributed to the observed differences of *indica* and *japonica* rice will need to be further investigated.

Three major hypotheses can explain LB at the *Pi-ta* locus: 1) Genes required for blast resistance are clustered together. This hypothesis is supported by the finding that *Pi-ta*-mediated resistance responses require another plant gene *Ptr(t)* and this gene has been mapped nearby the *Pi-ta* gene [25]. The *Ptr(t)* gene was recently fine mapped outside 800 kb of the *Pi-ta* gene (Wang and Jia, unpublished data) further suggesting LB can be reduced without selection for blast resistance. 2) The centromeric region of plant genome often has a significant amount of cross over suppression [30], [31], in fact, there is some recombination suppression at the centromeric region on chromosome 8 (Jia, unpublished data). Thus, the sizes of LB observed near the centromere may be larger than that in other regions of chromosome. 3) Incompatibility occurs between *indica* and *japonica* crosses. The fact that recombination suppression occurs in indica by japonica crosses but not indica by indica crosses indicate that large LB at the *Pi-ta* locus is mostly likely due to the incompatibility of *indica* and *japonica*. It would be a priority to investigate the size of LB involved in japonica parents in the present study with other japonica genotypes.

If large LB is due to the incompatibility between *indica* and *japonica*, perhaps genome sequence of parents in the present study should give us some ideas as what genes may be involved. The major homologous recombination gene DMC1 has types A and B [32], [33] in the reference genome Nipponbare. One member of the type A is in chromosome 12 [34]. Further sequence analysis of type A and functional validation will be needed to establish if type A plays any role in the observed LB between *indica* and *japonica* crosses. Additionally, genomic comparisons thus far have revealed that there are considerable differences on transposable elements on chromosome 12. Recently, a transposon at the promoter region of *Pi-ta* in resistant rice cultivars was found suggesting its active role in regulation of gene expression [17]. Predicted DNA sequences encode a protein with 844 amino acids with features similar in zinc fingers and transcription factors and with domains commonly found in hAT family dimerization (hATC) of a transposable element [17]. Additionally, a study at chromosome 8 suggested that transposable elements LTR-retrotransposon on chromosome 8 may play role in observed recombination suppression [35]. However, differences in number and type of transposable elements on chromosome 12 are not as great as that on chromosome 8. Further examination of recombination events of the crosses involved in rice cultivars that were not screened for *Pi-ta* mediated blast resistance, and sequence all genes on chromosome 12 in these cultivars may shed more light on the mechanisms of incompatibility between indica and japonica rice.

Understanding of LB on a particular chromosome is important for crop improvement. Rice cultivars with *Pi-ta* have been effective for preventing blast disease over two decades in the southern US [8], [9]. This could be due to LB at the *Pi-ta* region where all components including *Ptr(t)* [25] for blast resistance have been inherited together. Thus far, *Pi-ta* and *Pi-ta2* are the only blast *R* genes in that region that are effective at preventing blast disease in the US [10], [13], [36] although it is unclear if *Pi-ta2* is present in all *Pi-ta* containing cultivars in the southern US. Cloning of *Pi-ta2* should help to address this question. In contrast, LB can be a bottleneck for crop improvement. Genes for desired traits are often embedded with genes for unwanted traits which can be problematic for conventional crop improvement. In fact, historically it has been observed that yield potentials of 11 rice cultivars with *Pi-ta* were not as high as some rice cultivars without *Pi-ta* in Arkansas, suggesting the negative factors for yield may have been inherited from the original donor for *Pi-ta* due to LB at the *Pi-ta* locus. A population with mono- and digenic lines of *Pi-ta, Pi-ks/h* that are segregating for yield components [37] and near isogenic line with *Pi-ta* developed by Jia and Martin [25] should help us to determine epistatic relationship between blast resistance and yield potential in the future.

In summary, using five random mapping populations of rice we demonstrated that LB around a blast resistance gene that was largely due to the incompatibility between *indica* and *japonica* rice. Identified recombinant lines defined recombination suppression will be useful for further sequence, and analysis of molecular differences between *indica* and *japonica* rice in order to understand molecular basis of recombination suppression, a phenomena that has been commonly observed in plant genetics and breeding.

## Materials and Methods

### Choice of Rice Genotype (Table 2)

“Early” (RU9101001) is an early maturing, cold tolerant tropical *japonica* rice line developed by University of Arkansas (Moldenhauer, unpublished data). This line has been used as an experimental line for marker development and genetic studies. Katy (PI 527707), released in 1990, is a tropical *japonica* cultivar that contains *Pi-ta* [37] and has been a donor for *Pi-ta* for rice breeding programs in the Southern US. Lemont (PI 475833) is a tropical *japonica* cultivar grown from the 1980s to 1990s in the southern US that does not possess *Pi-ta* [38]. Jasmine 85 (PI 595927), a long-grain aromatic *indica* cultivar lacking *Pi-ta* initially developed at the International Rice Research Institute (IRRI), in the Philippines and adapted to the southern US, was officially released in 1989 by the USDA-ARS and the Texas Agricultural Experiment Station [39]. Cocodrie is a tropical *japonica* cultivar with good yield potential and lacks *Pi-ta* [40]. MCR 01-0277 (PI 641932) is a breeding line developed in the US that contains considerable *indica* genome [41]. Zhe733 (PI 629016) is an *indica* cultivar with high yield, early maturity and effective blast resistance genes lacking *Pi-ta* from China. “172” is the *indica* cultivar Amane (*Pi-ta/Pi-ta*) (PI 373335) from Sri Lanka that contains *Pi-ta,* but is susceptible to an avirulent isolate (Jia, unpublished data). It was predicted that Amane contains a defective component [*ptr(t)/ptr(t)*] required for *Pi-ta-*mediated disease resistance [21], [24]. The *indica* cultivar 172 contains *Pi-ta,* but is susceptible to the most of common races of blast [15] and was used as a parent crossed to Katy to develop a mapping population for detection of resistance to blast not due to *Pi-ta*, but due to the defect of additional critical component *Ptr(t)* [25]. A previous study genotyped all of these rice germplasm accessions, except MCR 01-0277, with 1536 SNP markers, and genomic contents of *indica* and *japonica* on rice chromosome 12 were verified by Zhao et al [42].

**Table 2:** Description of rice cultivars and breeding lines used in this study.^1^

Name	Plant Identification	Characteristics	Existence of *Pi-ta* 3	Reference
Early (RU9101001)	Unregistered	Tropical japonica line, early mature, blast susceptible	_	43
Katy	PI 527707	Tropical japonica cultivar withlarge introgression of indicafrom an indica landrace Tetep	+	37
Lemont	PI 475833	Tropical japonica cultivar	_	38
Jasmine 85	PI 595927	Indica	_	39
Cocodrie	PI 606331	Tropical japonica line, early mature		40
MCR01-0277	PI 641932	Indica like breeding line	_	41
Zhe 733	PI 629016	Early mature, blast resistant,high yielding indica	_	46, from China
“172”^2^	PI 37335	Indica	+	From Sri Lanka

1 Characteristics of rice genotypes were verified using 1536 SNP markers [42].

2 Cultivar 172 was susceptible to blast although it contains *Pi-ta* because lacking *Ptr(t)* [25].

3 Indicates the absence, and + indicates the presence of resistant *Pi-ta* allele in each genotype as determined using method described in [23].

### Mapping Populations

In order to reduce negative impact of recombination suppression and polymorphic marker density limitation of some parents, a total of five mapping populations derived from different indica and japonica and indica and indica crosses was used to investigate LB on rice chromosome 12. Five mapping populations, four of which were requested from the Genetic Stocks Oryza (GSOR) collection, USDA-ARS (http://www.ars.usda.gov/spa/dbnrrc/gsor) were used for this study: 1) Early/Katy: 87 individuals of an F_10_ recombinant inbred line (RIL) population derived from a cross of a tropical *japonica* line RU9101001 (*pi-ta/pi-ta*) with a tropical *japonica* cultivar Katy (*Pi-ta/Pi-ta*) [43]. About half (14 megabases) of chromosome 12 in Katy was derived from a landrace *indica* Tetep. 2) LJ: 93 individuals of an F_10_ RIL population derived from a cross of a tropical *japonica* Lemont (*pi-ta/pi-ta*) with an *indica* cultivar Jasmine 85 (*pi-ta/pi-ta*). 3) KZ: 89 individuals of an F_10_ RIL population of a cross of an *indica* Zhe733 (*pi-ta/pi-ta*) and tropical genetic stock *Kaybonnet-lpa1-1* (*Pi-ta/Pi-ta*) with the chromosome 12 identical to that in Katy [44]. 4) K172∶94 individuals of an F_2_ population derived from a cross of Katy [*Pi-ta/Pi-ta Ptr(t)Ptr(t)*] with ‘172’ [*Pi-ta/Pi-ta ptr(t)ptr(t)*] from Sri Lanka where both contain different *indica* origins for chromosome 12 This population was developed by the Molecular Plant Pathology program of DB NRRC (Jia, unpublished data). 5) MCR: 93 doubled haploid lines of the SB2 mapping population derived from a cross of a tropical *japonica* cultivar Cocodrie (*pi-ta/pi-ta*) and an *indica* derived germplasm MCR 01-0277 (*pi-ta/pi-ta*) [41].

### Plant Growth

Rice plants were grown in a greenhouse using sterilized local clay soils under conditions as previously described [45]. For RILs, seeds (5 to 10) from each line were directly sown on top of moistened soil in a small pot to produce 3 plants per line. Small pots with 4 holes at the bottom that allow roots to access water were placed in a tray with tap water. Leaves were bulked from 3 plants at the 3- to 4-leaf stage on ice for DNA extraction. For the F_2_ population, seeds were planted into a 64 well flat and tissue was harvested from each plant at the 3- to 4-leaf stage. DNA was prepared as described in Liu et al [46].

### SSR Analysis

To obtain dense coverage on chromosome 12, forty SSR markers were used to identify polymorphic markers from both parents and 28 polymorphic markers were identified for this study (Figure 2). The primer sequences and map position of the SSR markers were obtained from the Gramene Version 21 database (http://www.gramene.org). PCR reactions were performed in 25 µL reaction volumes consisting of 20 ng of DNA, 10 mM Tris–HCl pH 8.3, 50 mM KCl, 2.5 mM MgCl2, 300 nM of each primer, 1 U of Taq DNA polymerase (Promega, Madison, WI, USA).

For each marker analysis, the reverse primers were unlabeled to reduce the cost, and the forward primers were labeled with either 6FAM, PET, NED or Hex (Applied Biosystems, Foster City, CA, USA or Integrated DNA Technologies, Coralville, IA, USA). DNA were amplified with MJ Research Tetrad PCR machines (Waltham, MA, USA) under the following conditions: initial denaturation at 94°C for 5 min; then 30 cycles at 94°C for 30 s, 55–67°C (varies dependent on marker) for 30 s, and 72°C for 1 min; 5 min final extension at 72°C. PCR products were pooled based on size of amplified fragments (typically three markers per run along with LIZ-labeled size standard) to reduce cost, and the DNA was denatured by heating samples at 94°C for 5 min. The samples were separated on an ABI Prism 3730 DNA analyzer using the method as described by the manufacturer (Applied Biosystems). SSR fragment size was estimated using the software GeneMapper v 3.7 (Applied Biosystems).

### Blast Evaluation and Marker for *Pi-ta* Mediated Blast Resistance

Disease reaction to blast was determined using a modified standard pathogenicity assay as previously described in Jia et al [45]. Specifically, rice seedlings at 3–4 leaf stage in a plastic bag were spray-inoculated with blast spore suspension at 1–5×10^5^ spores/ML. After inoculation plastic bags were sealed to maintain high humidity for 24 h before removing out from bags. Subsequently, plants were maintained in a greenhouse for additional 6 days to allow the development of clear disease symptoms. The disease reaction was classified as 0–2 as resistance and 3–5 as susceptibility based on visual number and amount of lesions at the next of youngest leaf. In the K172 population F_2∶3_ progeny were also used to verify blast reactions [13] and marker segregation. SSR marker RM27941 in Katy was found to co-segregate perfectly with blast resistance [X. Wang and Y. Jia, (unpublished data)]. RM27941 was used as a marker for *Pi-ta* mediated blast resistance in the present study.

### Construction of Linkage Maps

JoinMap Version 4.0 was used for linkage analysis [47]. A minimum LOD score of 3.0 was used to identify groups and maps were generated using Maximum likelihood mapping with default settings. The order of the markers on each chromosome was referred to the SSR marker database of Cornell SSR 2001 (Cornell2001) as described in Gramene, and by Conaway-Bormanset et al [48] and Jia et al [23]. SSR marker data were converted for JoinMap and are summarized in the (Table S1, S2, S3, S4, S5, S6, S7, S8, S9, S10, Table S11).

## Supporting Information

Table S1 **Note to mapping population.**
(DOCX)

Table S2 **Mapping profile for Early Katy mapping population for JoinMap.**
(XLS)

Table S3 **SSR marker file for Early and Katy mapping population.**
(XLSX)

Table S4 **Mapping profile for LJ mapping population for JoinMap.**
(XLS)

Table S5 **SSR marker file for LJ mapping population.**
(XLS)

Table S6 **Mapping profile for Cocodrie and MCR mapping population for JoinMap.**
(XLS)

Table S7 **SSR marker file for Cocodrie and MCR mapping population.**
(XLS)

Table S8 **Mapping profile for Katy and172 mapping population for JoinMap.**
(XLS)

Table S9 **SSR marker file for Katy and 172 mapping population.**
(XLS)

Table S10 **Mapping profile for KZ mapping population for JoinMap.**
(XLS)

Table S11 **SSR marker file for Zhe733 and Katy mapping population.**
(XLS)
