# Supplementary material for: Indica and Japonica Crosses Resulting in Linkage Block and Recombination Suppression on Rice Chromosome 12
Source: PLoS One. 2012 Aug 17;7(8):e43066. doi: 10.1371/journal.pone.0043066 (PMC3422337; doi:10.1371/journal.pone.0043066)
Supplement: Table S1 — Note to mapping population. (DOCX) [file pone.0043066.s001.docx]

**Table S1.** Notes for mapping populations

| Mapping population | Unmapped SSR marker | One explaination |
| --- | --- | --- |
| RU9101001 X Katy | RM3483, RM19, RM1300 | recombination suppression |
| MCR X Cocodrie | RM28430, RM270, RM3739, and RM1300 | recombination suppression |
| Katy X 172 | RM19 | Too far from RM3246 |
